# Feasibility of high-resolution quantitative perfusion analysis in patients with heart failure

**DOI:** 10.1186/s12968-015-0124-2

**Published:** 2015-02-12

**Authors:** Eva Sammut, Niloufar Zarinabad, Roman Wesolowski, Geraint Morton, Zhong Chen, Manav Sohal, Gerry Carr-White, Reza Razavi, Amedeo Chiribiri

**Affiliations:** King’s College London BHF Centre of Excellence, NIHR Biomedical Research Centre and Wellcome Trust and EPSRC Medical Engineering Centre at Guy’s and St. Thomas’ NHS Foundation Trust, Division of Imaging Sciences and Biomedical Engineering, The Rayne Institute, St. Thomas’ Hospital, London, UK; Department of Cardiology, Guy’s and St Thomas’ Hospital, London, UK; Division of Imaging Sciences and Biomedical Engineering, King’s College London, 4th Floor North Wing, St Thomas’ Hospital, SE1 7EH London, UK

**Keywords:** Heart failure, Quantitative perfusion, Cardiovascular magnetic resonance, Gadolinium

## Abstract

**Background:**

Cardiac magnetic resonance (CMR) is playing an expanding role in the assessment of patients with heart failure (HF). The assessment of myocardial perfusion status in HF can be challenging due to left ventricular (LV) remodelling and wall thinning, coexistent scar and respiratory artefacts. The aim of this study was to assess the feasibility of quantitative CMR myocardial perfusion analysis in patients with HF.

**Methods:**

A group of 58 patients with heart failure (HF; left ventricular ejection fraction, LVEF ≤ 50%) and 33 patients with normal LVEF (LVEF >50%), referred for suspected coronary artery disease, were studied. All subjects underwent quantitative first-pass stress perfusion imaging using adenosine according to standard acquisition protocols. The feasibility of quantitative perfusion analysis was then assessed using high-resolution, 3 T *kt* perfusion and voxel-wise Fermi deconvolution.

**Results:**

30/58 (52%) subjects in the HF group had underlying ischaemic aetiology. Perfusion abnormalities were seen amongst patients with ischaemic HF and patients with normal LV function. No regional perfusion defect was observed in the non-ischaemic HF group. Good agreement was found between visual and quantitative analysis across all groups. Absolute stress perfusion rate, myocardial perfusion reserve (MPR) and endocardial-epicardial MPR ratio identified areas with abnormal perfusion in the ischaemic HF group (p = 0.02; p = 0.04; p = 0.02, respectively). In the Normal LV group, MPR and endocardial-epicardial MPR ratio were able to distinguish between normal and abnormal segments (p = 0.04; p = 0.02 respectively). No significant differences of absolute stress perfusion rate or MPR were observed comparing visually normal segments amongst groups.

**Conclusions:**

Our results demonstrate the feasibility of high-resolution voxel-wise perfusion assessment in patients with HF.

## Background

Despite recent advances in diagnosis and management, heart failure (HF) remains a common cause of death and morbidity [1-3]. Myocardial ischemia is known to be one of the proposed pathophysiological mechanisms of HF and it is increasingly evident that the management of ischemia represents a potential therapeutic target for some of these patients [4-6].

In recent years, cardiovascular magnetic resonance (CMR) has established itself as an important component to the assessment and management of patients with coronary artery disease (CAD) and HF [7,8]. CMR is considered the reference method for the assessment of global and regional ventricular function and left ventricular wall mass [9]. CMR has become a robust and widely-available tool for assessment of ischaemic scar burden and myocardial perfusion imaging in patients with CAD [10-12] and can be used to help elucidate aetiology of HF [13]. However, there are no data currently available on the feasibility of perfusion CMR in patients with HF. The evaluation of myocardial perfusion status in patients with HF can be challenging due to LV remodelling and wall thinning, the presence of scar, and respiratory artefacts. 3 Tesla (3 T) *kt* CMR perfusion enables high spatial resolution perfusion assessment and provides data suitable for voxel-wise quantitative analysis [14,15]. The availability of combined methods for high-resolution imaging and voxel-wise quantitative assessment may enable the use of perfusion CMR to assess myocardial perfusion status also in patients with dilated and remodelled ventricles, although the evidence for this is lacking. Therefore, the aim of this study was to test the feasibility of 3 T *kt* high-resolution and voxel-wise quantitative perfusion CMR in this subgroup of patients.

## Methods

We identified patients referred for perfusion CMR to assess HF aetiology. Patients were then retrospectively classified on the basis of the aetiology of HF as ischaemic (LVEF < 50% with scar arising from the subendocardium or transmural in a location corresponding to a coronary territory) or non-ischaemic (LVEF < 50%, either no scar or mid-myocardial or epicardial scar with invasive coronary angiogram confirming unobstructed epicardial coronary arteries) [13]. A group of patients with normal LV function (LVEF >50%) referred for suspected coronary artery disease were studied as control subjects.

All patients studied provided written consent (09/H0802/78) and the study was conducted in accordance with the Declaration of Helsinki.

Images were acquired on a Philips Achieva 3 T (TX) system, equipped with a 32-channel cardiac phased array receiver coil (Philips, Best, the Netherlands). First pass perfusion imaging consisted of a high-resolution *kt* turbo-gradient echo sequence (imaging parameters: shortest echo time (range 1.35 to 1.54 ms), shortest repetition time (range 2.64 to 3.12 ms), 18° flip angle, 90° saturation prepulse, 120 ms prepulse delay, typical TR 2.6 ms, typical TE 0.9 ms, typical spatial resolution 1.2×1.2×10mm. Three short-axis slices (basal, mid and apical) were acquired over every heartbeat covering 16 of the standard myocardial segments (segment 17 was excluded). Stress imaging preceded rest imaging by 14 ± 2 min (range 10 to 19 min). For stress imaging, 140 μg/kg/min of adenosine was administered intravenously for 4 min. Imaging commenced 3 min into the infusion and continued for 1 min during the acquisition of the images. All subjects were asked to abstain from caffeine and caffeine-containing food and drink for at least 24 hours before the scan, according to institutional practice.

Perfusion data were acquired during first pass injection of 0.075 mmol/kg Gadobutrol (Gadovist, Schering, Germany) at 4 ml/minute followed by a 20 ml saline flush. A dual bolus contrast agent scheme was used to correct for signal saturation of the arterial input function as previously described [16,17]. A correction map was created from a proton density-based image based on the same projections as the perfusion scans for correction of spatial inhomogeneities due to surface coils [18]. The CMR scan protocol included late gadolinium enhancement imaging for all subjects after a top up dose of contrast agent to a total dose of 0.2 mmol of gadolinium/kg of body weight.

### Visual analysis

The studies were analyzed visually by two independent experts blinded to all other data. CMR scans were classified visually positive for ischemia in the presence of a perfusion defect (>60 degrees in either the basal or mid-ventricular slices, or >90 degrees in the apical slice) which was transmural, or involving ≥2 adjacent myocardial segments according to the criteria by Hussain et al. [16]. In the case of disagreement between observers, the images were reviewed together, and a consensus was reached. Late gadolinium enhancement and rest perfusion scans were then used to differentiate between stress-induced perfusion abnormalities and scar-related perfusion defects.

### Quantitative analysis

An experienced operator, blinded to visual assessment and other clinical data, performed quantitative analysis using software and methods developed and previously validated against phantom and PET data [19,20]. The implementation of high-resolution signal intensity (SI) analysis required accurate respiratory motion correction and myocardial contour delineation. Respiratory motion was corrected using affine image registration by maximization of the joint correlation between consecutive dynamics within an automatically determined region of interest. A temporal maximum intensity projection was calculated to serve as a feature image for an automatic contour delineation method. The operator then manually optimized the automatically generated contours to avoid partial volume effects at the endocardial and epicardial border as previously described [21]. Areas of subendocardial dark-rim artefact occurring at the arrival of the main bolus of contrast agent in the LV were carefully excluded from the segmentation.

Quantitative perfusion analysis was performed by Fermi deconvolution according to the methods described by Wilke et al. [22] and Jerosch-Herold et al. [23] where time curves for the tissue impulse response function, *h(t)*, were fitted to the Fermi function with the following analytical expression:

$$ h(t)=R\left[\frac{1}{{}_e\left(t-{\tau}_0-{\tau}_d\right){k}_{+1}}\right]u\left(t-{\tau}_d\right) $$

using a Marquardt-Levenberg nonlinear least square algorithm by letting *k*, *R* and *τ*_0_ vary and keeping *τ*_*d*_ fixed. In the above equation, *u*(*t*–*τ*_*d*_) is the unit step function. The *τ*_*d*_ accounts for the delay time between the appearance of the signal in the LV blood pool and myocardial region of interest (ROI) [24]. *τ*_0_ characterizes the width of the shoulder of the Fermi function during which little or no contrast agent had left the ROI. *R* is the index of contrast agent influx parameter and *k* represents the decay rate of *h*(*t*) due to contrast agent washout. Using the above equation, myocardial blood flow (MBF) estimates are calculated as *h*(*t*) at *t*=0 [25]. Myocardial perfusion reserve (MPR) was calculated from division of stress perfusion rate by rest perfusion rate.

In addition, voxel-wise MPR results from the epicardial half (outer 50% transmural thickness) and from the endocardial half of each segment (inner 50% transmural thickness) were averaged and the endocardial to epicardial MPR ratio (endocardial MPR/epicardial MPR) was calculated.

### Coronary angiography

Invasive coronary angiogram was performed on all patients with ischaemic cardiomyopathy or normal LV function within 6 months of the CMR scan.

### Image quality

A visual score was given for image quality of each dataset using a four-point scale: 1—poor, 2—fair, 3—good, and 4— excellent. The severity of respiratory artefacts and dark rim artefacts were also scored on a four-point and three-point scale respectively. For respiratory artefacts: 1 – non-diagnostic; 2 – severe artefacts but diagnostic; 3 – mild artefacts; 4 – no artefacts. For dark rim artefacts: 1- circumferential; 2- segmental; 3- absent.

### Statistical analysis

Data are presented as mean and standard deviation. Group means were compared using paired and unpaired Student *t* test and 1-way ANOVA as appropriate. The statistical analyses were performed using PASW software for Macintosh (IBM, Chicago, Illinois, version 21). Agreement between perfusion CMR and coronary angiography for the diagnosis of CAD was evaluated using a Kappa agreement test. Mann-Whitney and Chi-squared tests were used to test the qualitative measurements for statistical significance, with a p value threshold of 0.05.

## Results

A total of 91 subjects were studied. This included 58 patients with HF (LVEF 39 ± 10%) and 33 patients with normal LV function (Normal LV group) (LVEF 64 ± 5%). In the HF group, 30 patients (51.7%) had a final diagnosis of ischaemic cardiomyopathy (ICM) and the remaining 28 subjects had a final diagnosis of non-ischaemic cardiomyopathy (NICM). Demographics, CMR structural findings and haemodynamic parameters are given in Table 1.

**Table 1:** **Table showing demographic data and structural CMR findings for subjects studied**

	**NICM group**	**ICM group**	**Normal LV (NLV) group**	**P value NICM vs ICM group**	**P value NICM vs NLV group**	**P value ICM vs NLV group**	**P value between all groups**
Male Gender	19/28 (68%)	22/30 (73%)	22 (67%)	-	-	-	-
Age (years)	58 ± 14	63 ± 12	54 ± 18	0.78	0.77	0.08	0.79
BSA (m^2^)	1.86 ± 0.21	1.96 ± 0.19	1.90 ± 0.19	0.27	1.00	0.70	0.27
LV EDV (ml/m^2^)	102 ± 28	112 ± 33	71 ± 18	0.48	<0.0001*	<0.0001*	<0.0001*
LV ESV (ml/m^2^)	61 ± 26	73 ± 31	25 ± 8	0.16	<0.0001*	<0.0001*	<0.0001*
LVEF (%)	42 ± 9	37 ± 10	65 ± 4	0.06	<0.0001*	<0.0001*	<0.0001*
LV mass (g/m^2^)	73 ± 18	70 ± 18	55 ± 13	1.00	0.001*	0.006*	0.001*
RV EDV (ml/m^2^)	80 ± 22	74 ± 20	72 ± 18	0.88	0.39	1.00	0.30
RV ESV (ml/m^2^)	40 ± 20	35 ± 16	28 ± 10	0.54	0.008*	0.24	0.01*
RVEF (%)	51 ± 13	55 ± 12	62 ± 7	0.58	0.001*	0.02*	0.001*
LA area (cm^2^)	25 ± 7	26 ± 6	22 ± 5	1.00	0.37	0.07	0.07
RA area (cm^2^)	21 ± 6	21 ± 5	19 ± 4	1.00	0.77	0.76	0.43
Heart rate (bpm)							
Stress	103 ± 4	104 ± 3	117 ± 7	0.06	<0.0001*	<0.0001*	
Rest	62 ± 5	64 ± 2	75 ± 3	0.06	<0.0001*	<0.0001*	
SBP (mmHg)							
Stress	102 ± 6	102 ± 4	110 ± 8	0.73	<0.0001*	<0.0001*	
Rest	111 ± 3	109 ± 3	126 ± 7	0.09	<0.0001*	<0.0001*	
DBP (mmHg)							
Stress	69 ± 5	69 ± 5	76 ± 3	0.43	<0.0001*	<0.0001*	
Rest	73 ± 4	73 ± 5	79 ± 4	0.54	<0.0001*	<0.0001*	

(Abbreviations: SBP- systolic blood pressure; DBP-diastolic blood pressure).

*Denotes significance.

### Visual analysis

In the ICM group, 25/30 (83%) patients showed evidence of a perfusion abnormality. Of these 19/25 (76%) patients showed stress-induced perfusion abnormalities, either extending beyond an area of scar or in an area served by a separate coronary territory. On segmental analysis, there were 73 segments showing stress-induced perfusion abnormalities (4.06 ± 2.05/patient). 90 segments showed scar-related perfusion abnormalities (4.29 ± 2.13/patient).

In the NICM group, no visual perfusion abnormalities were reported and 7/28 patients (for a total of 25 segments) showed late enhancement with a non-ischaemic-pattern [13].

15/33 (46%) patients in the Normal LV group showed stress-induced perfusion abnormalities, with a total number of 105 positive segments (6.4 ± 4.5/patient). No patients from the Normal LV group showed scar on late gadolinium enhancement images.

### Quantitative analysis

#### Stress and rest perfusion estimates

Detailed results of stress and rest quantitative analysis are reported in Table 2.

**Table 2:** **Table showing rest, stress and MPR perfusion values for groups studied**

	**Visually normal segments**	**Visually abnormal, non-scarred segments**	**Visually abnormal, scarred segments**	**p-value Normal vs non-scarred segments**	**p-value Normal vs scarred segments**
Stress perfusion Normal LV group (ml/g/min)	2.3 ± 1.3	1.6 ± 0.8	-	0.08	-
Stress perfusion NICM group (ml/g/min)	1.9 ± 0.8	-	-	-	-
Stress perfusion ICM group (ml/g/min)	2.2 ± 1.0	1.8 ± 0.9	1.5 ± 0.7	0.02*	0.006*
Rest perfusion Normal LV group (ml/g/min)	0.86 ± 0.30	0.9 ± 0.4	-	0.83	-
Rest perfusion NICM group (ml/g/min)	0.90 ± 0.19	-	-	-	-
Rest perfusion ICM group (ml/g/min)	1.0 ± 0.4	1.0 ± 0.4	0.9 ± 0.4	0.25	0.83
MPR Normal LV group	2.6 ± 1.1	1.7 ± 0.8	-	0.04*	-
MPR NICM group	2.2 ± 0.8	-	-	-	-
MPR ICM group	2.3 ± 0.8	1.8 ± 0.9	1.7 ± 0.7	0.04*	0.004*

*Denotes significance.

No significant difference in visually normal segments was observed between groups, with an average stress perfusion rate of 2.2 ± 1.0 ml/g/min in the ICM group, 1.9 ± 0.8 ml/g/min in the NICM group and 2.3 ± 1.3 ml/g/min in the Normal LV group (p = 0.18).

In the ICM group, there was a significant difference in stress perfusion rate between visually normal and abnormal segments (2.2 ± 1.0 ml/g/min vs 1.8 ± 0.9 ml/g/min, p = 0.02). The presence of ischaemic scar was associated with significantly reduced perfusion rate compared to visually normal segments (1.5 ± 0.8 ml/min vs 2.2 ± 1.0 ml/g/min; p = 0.005).

Within the Normal LV group, the difference in stress perfusion rate between visually normal and abnormal segments was less pronounced and not statistically significant (2.3 ± 1.3 ml/g/min vs 1.6 ± 0.8 ml/g/min, p = 0.08).

There was no significance difference in stress perfusion values between the ICM and Normal LV groups in visually abnormal segments (p = 0.47).

**Figure 1:**
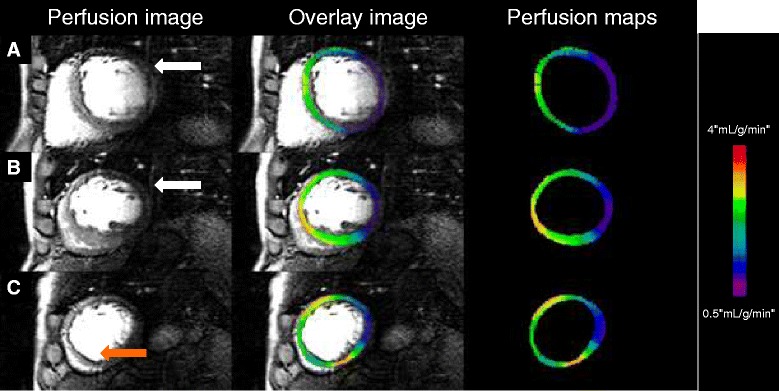
**Perfusion images from a HF patient with a circumflex coronary artery lesion causing a perfusion abnormality extending from base (A) to mid (B) to apical (C) level.** Images seen vertically on the right show the perfusion maps corresponding to the ventricular level, the central column shows superimposed images. Note that there is mild respiratory artifact (white arrows) and that dark rim artifact seen in the apical inferoseptal wall (orange arrow).

**Figure 2:**
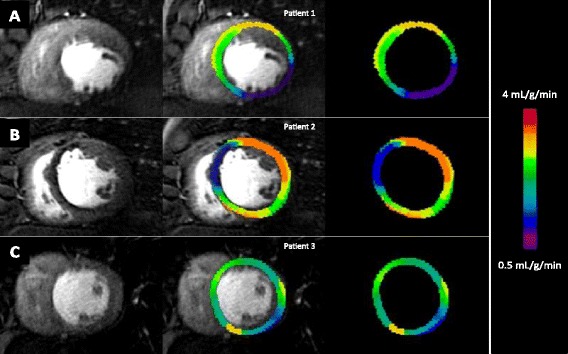
**Perfusion images from three different HF patients with image on the left, perfusion map on the right and superimposed images in the centre.** Row **A** shows thinning of the mid inferior wall with peri-infarct ischemia in the territory of the RCA. Row **B** shows ischemia in the territory of the LAD. Row **C** shows homogenous perfusion in a patient with thinned and dilated ventricle.

Examples of high-resolution, voxel-wise perfusion maps are given in Figures 1 and 2.

#### MPR

Detailed results of MPR analysis are also reported in Table 2.

No significant difference in visually normal segments was observed between groups, with an average MPR of 2.3 ± 0.8 in the ICM group, 2.2 ± 0.9 in the NICM group and 2.6 ± 1.1 in the Normal LV group (p = 0.19).

In the ICM group, there was a significant difference in MPR between visually normal and abnormal segments (2.3 ± 0.8 vs 1.8 ± 0.9, p = 0.04). The presence of ischaemic scar was associated with significantly reduced perfusion rate compared to visually normal segments (1.68 ± 0.08 vs 2.3 ± 0.8, p = 0.004).

Similarly, within the Normal LV group, the difference in MPR between visually normal and abnormal segments was statistically significant (2.6 ± 1.1 vs1.7 ± 0.8, p = 0.04).

There was no significance difference in MPR values between the ICM and Normal LV groups in visually abnormal segments (p = 0.62).

#### Endocardial-epicardial MPR ratio

There was no significant difference in the endo-epi MPR ratio in visually normal segments across groups with an endo-epi MPR ratio of 1.05 ± 0.16 in ICM patients, 1.02 ± 0.04 in NICM patients and 1.00 ± 0.06 in Normal LV patients (p = 0.57).

There was a significant difference in endo-epi MPR ratio between visually normal and visually abnormal non-scarred segments in both the ICM and Normal LV groups (ICM group: 1.04 ± 0.09 versus 0.96 ± 0.12 respectively, p = 0.02; Normal LV group: 1.00 ± 0.05 versus 0.93 ± 0.06 respectively, p = 0.02).

### Coronary angiography

The results of invasive coronary angiography and perfusion CMR in relation to coronary artery territories, including Kappa results are detailed in Table 3.

**Table 3:** **Table showing results of coronary angiography versus perfusion CMR results for patients in ICM and Normal LV groups**

	**LAD territory**				**CX territory**				**RCA territory**			
**Group**	**CAD on angio**	**LGE only**	**iPD (no LGE/beyond LGE)**	**Kappa (significance)**	**CAD on angio**	**LGE only**	**iPD (no LGE/beyond LGE)**	**Kappa (significance)**	**CAD on angio**	**LGE only**	**iPD (no LGE/beyond LGE)**	**Kappa (significance)**
ICM group	25/30 (83%)	12/25 (48%)	11/25 (44%)	0.462 (p = 0.003*)	11/30 (37%)	4/11 (36%)	4/11 (16%)	0.603 (<0.001*)	13/30 (43%)	9/13 (69%)	6/13 (46%)	0.718 (p = <0.001*)
Normal LV group	16/33 (48%)	0/33	6/16 (37.5%)	0.382 (0.005*)	11/33 (33%)	0/11	6/11 (55%)	0.615 (<0.001*)	13/33 (39%)	0/13	6/13 (46%)	0.510 (p = 0.001*)

(Abbreviations: angio - invasive coronary angiogram; CAD - coronary artery disease; iPD- inducible perfusion defect; LAD- left anterior descending artery; Cx- circumflex artery; RCA- right coronary artery, LGE-late gadolinium enhancement).

*Denotes significance.

### Image quality

**Figure 3:**
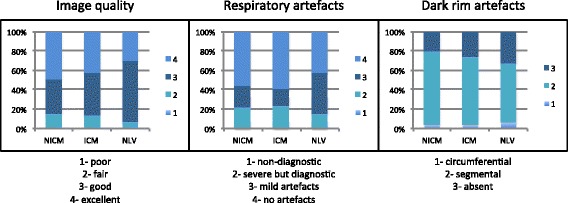
**Figure showing the relative scores for image quality, respiratory artefacts and dark rim artefacts according to group.**

Detailed results of overall image qualitative assessment, respiratory artefacts and dark rim artefacts are presented in Table 4 and Figure 3, with no significant differences observed between groups. The average angular extent of dark rim artefact was 27° (range 9-41°).

**Table 4:** **Table showing results of the Chi-squared tests for qualitative assessment of image quality showing no significant differences between groups**

	**Chi-square**	**P value**
Overall image quality	2.559	0.6342
Respiratory artefacts	5.359	0.2524
Dark rim artefacts	1.863	0.7609

## Discussion

This study has several novel findings. Most importantly, it demonstrates the feasibility of quantitative first-pass perfusion analysis in patients with heart failure using a combination of high-resolution *kt* 3 T acquisition methods and high-resolution voxel-wise quantification. There were significant differences of MPR between visually normal and abnormal segments in both ICM and Normal LV groups. In the ICM group stress perfusion values were also statistically significant between visually normal and abnormal segments. However, this was not the case in the Normal LV group. This could be explained by the relatively low number of subjects positive for ischemia included in the study and is in keeping with previous validation studies that compared CMR perfusion and PET, showing good correlation for MPR values and weaker correlation for absolute stress perfusion values. This may relate to differences in the methods used for quantification such as the tracer properties, model assumptions, fitting methods and parameter constraints. MPR cancels out a degree of variability and this may help explain our results [26].

CMR is now well established for the evaluation of patients with coronary disease and heart failure. Perfusion CMR is increasingly used to determine aetiology of heart failure and to plan patient management. At the same time, CMR is now well established for the evaluation of patients with CAD and ischemia detection is rapidly becoming one of the main indications for CMR examination [27]. Studies have also shown that even in non-ischaemic cardiomyopathy the presence of ischemia has implications for the occurrence of adverse events [5,6,28].

Perfusion CMR has been previously shown to be at least as accurate as nuclear perfusion imaging in patients with angina [12,29,30] and quantitative perfusion CMR has been validated against FFR [31,32], microspheres [22,33], Single Photon Emission Computed Tomography (SPECT) [34] and Positron Emission Tomography (PET) [20].

Within the field of perfusion CMR, several technical developments have refined the sequences. The use of an advanced sequence such as *kt* sensitivity encoding allows considerable improvement in spatial resolution with improved image quality, signal-to-noise, contrast-to-noise and reduction in the transmural extent of dark-rim artefacts, particularly at 3 T [15]. The advantages of the *kt* sequence in comparison with standard perfusion CMR sequences when using visual assessment has been demonstrated in patients with normal LV function [14,35].

To our knowledge, there are no previous studies focusing on perfusion in patients with heart failure using CMR. Only a few studies, using PET, have been performed specifically exploring perfusion abnormalities in patients with HF and have produced conflicting results [36-38]. Van Den Heuvel et al. studied a group of NICM patients using PET and showed a similar perfusion rate at rest but reduced global myocardial perfusion reserve (MPR) versus a group of healthy controls [36]. In contrast, Neglia et al. showed a reduced MPR at rest as well as during pacing and pharmacological stress in patients with NICM [38]. The concept that even in NICM, the presence of silent ischemia may contribute to progressive impairment of LV function was supported by findings by a study by Tio et al. patients with NICM were assessed with PET and dobutamine stress [39]. They found that MPR was significantly higher in segments with higher contractile reserve and lower in the segments which did not change or deteriorated with stress.

Non-invasive assessment of myocardial perfusion in patients with HF can be challenging for a number of reasons. Foremost, in cases of advanced HF the LV wall is often thinner and remodeled, requiring higher spatial resolution to reliably identify subendocardial areas of stress-induced ischemia or areas of peri-infarct ischemia. PET is considered the reference standard for in-vivo quantitative perfusion assessment. However, it is limited by the intrinsically lower spatial resolution and this might become an important limiting factor for the assessment of dilated and remodelled ventricles.

Perfusion CMR, and in particular 3 T kt, offers higher spatial resolution and arguably is best placed to identify areas of ischemia in these patients. In addition, the advantage of CMR over other techniques to directly visualise scar enables more precise assessment of peri-infarct ischemia. This reduces the risk of including scarred areas in segmental averages of perfusion. Perfusion rate in areas of scar was found to be reduced which, if included in the analysis, could result in false positive ischemia results.

We observed a non-significant trend towards lower perfusion rates in visually normal segments of patients with NICM, which would be in keeping with the results previously obtained by PET. However, this comparison is beyond the scope of the study and would need to be addressed in future work.

Importantly, our results demonstrate a significant endocardial-epicardial MPR ratio in ischaemic segments, even in patients with HF who have thinned and remodelled ventricles. This is in keeping with findings of a study by Parodi et al. where microspheres were injected into hearts of patients at time of transplantation, demonstrating the presence of a perfusion gradient in the presence of extensive LV remodelling [40]. To our knowledge, our data are the first to show that non-invasive quantification of myocardial perfusion in multiple independent layers of myocardium is feasible also in this group of patients.

Perfusion assessment in patients with HF can be further complicated by a well-recognised irregularity in respiratory motion and reduced tolerance to breath-holding which can result in a reduction in image quality [41-43]. These respiratory artefacts are likely to affect *kt* sequences, used in this study, more significantly than other perfusion sequences. We did not observe this effect in our study, which may be a reflection of good patient coaching and careful selection of the time for the breath hold command.

### Limitations of the study

1. This is a feasibility study. No reference standard except for visual assessment was used. However, visual assessment is considered the clinical diagnostic standard for perfusion CMR evaluation and extensive validation is available against Fractional Flow Reserve (FFR), invasive angiography, Single Positron Emission Computed Tomography (SPECT) and PET.
2. Though we were able to compare our findings with invasive coronary angiography, this serves as a limited reference method in the absence of invasive functional assessment of coronary flow reserve. Furthermore, the variability in coronary anatomy in terms of territory supplied, may underestimate the degree of correlation between the two techniques.
3. Results might not be easy to generalise to other CMR sequences and field-strengths, or to standard (segmental) quantitative analysis methods. The quantification process may need to be adjusted for other field strengths and perfusion sequences and this could form part of future work.

## Conclusion

This study demonstrates that the combination of 3 T *kt* high-resolution perfusion CMR with high-resolution voxel-wise quantitative analysis appears feasible in this group of heart failure patients. This approach allows additional information gained from improved spatial resolution to be preserved, a feature that is of crucial importance in thinned and remodeled ventricles.
